# Influence of Morphological Characteristics of Coarse Aggregates on Skid Resistance of Asphalt Pavement

**DOI:** 10.3390/ma16144926

**Published:** 2023-07-10

**Authors:** Yuanshuai Dong, Zihao Wang, Wanyan Ren, Tianhao Jiang, Yun Hou, Yanhong Zhang

**Affiliations:** 1 China Highway Engineering Consulting Group Co., Ltd., No. 17 Changyungong Road, Haidian District, Beijing 100089, China; 2 Research and Development Center on Highway Pavement Maintenance Technology, CCCC, No. 116 Zizhuyuan Road, Haidian District, Beijing 100097, China; 3 Research and Development Center of Transport Industry of Technologies, Materials and Equipments of Highway Construction and Maintenance, No. 116 Zizhuyuan Road, Haidian District, Beijing 100097, China; 4 School of Civil and Transportation Engineering, Beijing University of Civil Engineering and Architecture, No. 1 Zhanlanguan Road, Xicheng District, Beijing 100044, China; 5 Collaborative Innovation Center of Energy Conservation & Emission Reduction and Sustainable Urban-Rural Development in Beijing, No. 1 Zhanlanguan Road, Xicheng District, Beijing 100044, China; 6 China Civil Engineering Construction Corporation, No. 4 Beifengwo Road, Haidian District, Beijing 100038, China

**Keywords:** actual pavement, skid resistance, coarse aggregate, morphological characteristics, attenuation characteristics, grey correlation entropy analysis

## Abstract

This research aims to improve the durability of skid resistance of asphalt pavement from the perspective of coarse aggregates based on on-site investigation. Firstly, the skid resistance of six representative actual roads was tested during two years by employing the Dynamic Friction Tester and the attenuation characteristics of skid resistance of different types of asphalt pavements were analyzed. Secondly, core samples were drilled onsite and coarse aggregates were extracted from the surface layer of the core samples. The morphological parameters of coarse aggregates were collected by a “backlighting photography” system and three-dimensional profilometer, and the variation rules of angularity and micro-texture of coarse aggregates were investigated. Finally, the correlation between the morphological characteristics of coarse aggregates and the pavement skid resistance was established based on the grey correlation entropy. The research results show that with the increase in service time, the attenuation rate of skid resistance of asphalt pavement gradually slows down; the angularity of coarse aggregates gradually decreases, and the micro-texture on the wearing surface gradually wears away. The grey correlation entropy between all the micro-texture indexes of coarse aggregates and dynamic friction coefficient, as well as between the roundness and skid resistance is more than 0.7, whereas the correlation between other evaluation indicators and the dynamic friction coefficient is poor, indicating that compared with the angularity of coarse aggregates, the micro-texture affects the skid resistance of actual asphalt pavement more greatly. In engineering applications, the use of coarse gradation, coarse aggregates with high roughness or high anti-wear performance can slow down the attenuation of pavement skid resistance, so that the pavement can maintain superior long-term anti-skidding performance.

## 1. Introduction

Traffic safety issues have always been a common challenge faced by countries around the world, and insufficient skid resistance of pavement is one of the main factors leading to frequent road traffic accidents [1,2,3,4]. In essence, excellent pavement skid resistance is ensured by good and stable macro- and micro-textures on its surface layer. The surface texture with a wavelength range of 0.5–50 mm is referred to as macro-texture, while the wavelength range of 0–0.5 mm is referred to as micro-texture. They affect the skid resistance of the vehicle when driving at high and low speeds, respectively, and sufficient friction cannot be generated by the contact between the tire and pavement in the case of too scarce pavement surface texture [5,6]. For this reason, in recent years, many scholars at home and abroad have carried out extensive research on the surface texture and anti-skidding performance of asphalt pavement. For instance, the skid resistance of pavement, especially in wet road conditions on rainy days, is closely related to macro-texture which provides a drainage system for water on the pavement surface, thus preventing a buildup of water between the tire and the pavement and resultant hydroplaning [7,8,9]. In the meantime, researchers believe that the type, apparent morphology and mineral composition of aggregates in the surface layer of pavement play an important role in the formation of surface texture [5,10,11,12,13]. Based on the skid resistance measurement results in the laboratory, researchers have pointed out that the anti-skidding performance of asphalt pavement depended on the effective friction and texture depth of the pavement [14,15,16,17]. Besides, using steel slag and other materials with high wear resistance as the aggregates to prepare asphalt mixtures or construct asphalt pavement can also improve the skid resistance [18,19,20]. By conducting laboratory accelerated loading tests on asphalt mixture specimens prepared with different aggregates, the results revealed that aggregates such as basalt and granite could provide excellent long-term pavement skid resistance for pavement [21,22,23]. In addition, different levels of abrasion tests on coarse aggregates have been carried out in the laboratory, and the correlation between their surface texture and the skid resistance has been analyzed [24,25]. The research results showed that the surface texture features of aggregates have a significant impact on long-term pavement skid resistance [26]. Moreover, the mineral composition, wear resistance and surface texture of various aggregates have been investigated, and the results demonstrate that the mineral hardness of aggregates has a great impact on their wear resistance [11,27,28,29,30,31]. Furthermore, by conducting accelerated loading tests on asphalt mixtures prepared with different aggregates, the results revealed that improving the wear resistance of aggregates could help reduce the attenuation rate of the skid resistance of asphalt pavement.

In summary, although many scholars at home and abroad have conducted in-depth research on the skid resistance of asphalt pavement, most of them are carried out in the laboratory, and there is a lack of studies based on the skid resistance of actual pavements, especially for morphological features of coarse aggregates. In this case, the environment, as a key factor affecting the skid resistance deterioration, has not been fully considered. At the same time, the wear effect of vehicle loads on pavement surfaces is different from that of laboratory-accelerated loading equipment. During the construction and operation stages of asphalt pavement, the skid resistance varies significantly among different actual asphalt pavements due to differences in pavement material properties and construction conditions, as well as factors such as changes in the operating environment. Therefore, this study aims to explore the attenuation law of skid resistance of different types of asphalt pavements through long-term tracking of skid resistance of actual roads under real service conditions, and to analyze the correlation between morphological features of coarse aggregates in the surface layer and skid resistance of actual pavements, thus contributing to the evaluation of aggregate morphological features and improvement in skid resistance.

## 2. Research Methods

### 2.1. Test Roads

The test roads selected are all located in Beijing, including Shidan (SD) Road, Shuinan (SN) Road, Luanchi (LC) Road, Yangyan (YY) Road, Xiyuan (XY) Road and Shunping (SP) Side Road. The basic information of the test roads is shown in Table 1, and the mixture composition information is shown in Table 2. Among the six roads, Shidan road is a newly constructed road while all the other five roads are put into service after major repair or upgrading projects and they have been milled and repaved with surface layers. Limestone is used in most of the roads in Beijing, so only one road with basalt was selected.

### 2.2. Measurement Time and Positions

The selected six roads were basically completed in August 2019. Their pavement skid resistance was tracked during the two years from opening to traffic to September 2021. During the two years, the pavement skid resistance was tested four times, November 2019, September 2020, December 2020 and September 2021, respectively, and set as stages I, II, III and IV. According to the Chinese Specification JTG 3450-2019 [32], the measurement and sampling positions were determined according to the uniform method in T0902-2019, and the measurement and core-drilling positions for the subsequent test stage the latter three measurement stages were kept to approach the initial measuring position in stage I.

### 2.3. Measurement Methods

#### 2.3.1. Measurement of Pavement Skid Resistance

**Figure 1:**
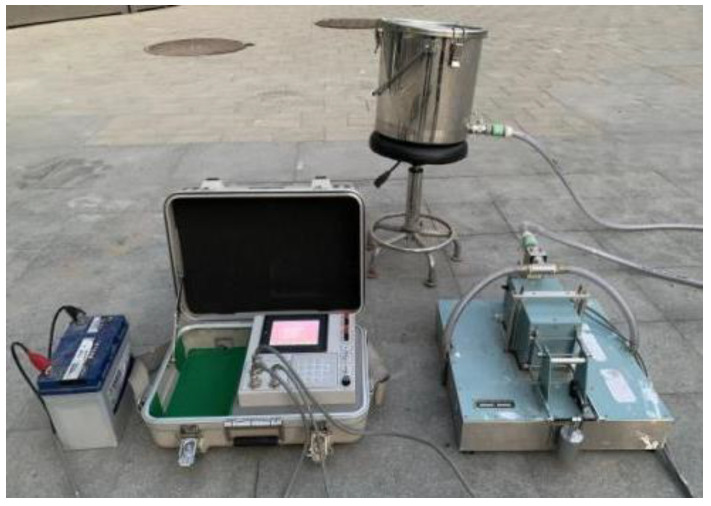
Dynamic Friction Tester.

The pavement skid resistance was measured by the Dynamic Friction Tester (DFT) (as shown in Figure 1) according to T0968-2008 in the Chinese Specification JTG 3450-2019 [32]. The Dynamic Friction (DF) can be measured by the DFT to characterize the skid resistance of vehicles at various speeds within the range of 0~80 km/h in steps of 1 km/h. For actual roads, the pavement skid resistance when vehicles are running at a high speed is usually characterized by DF_60_, namely the pavement DF coefficient at 60 km/h. Hence, DF_60_ was employed as the representative value of pavement skid resistance.

#### 2.3.2. Acquisition Method of Coarse Aggregates

**Figure 2:**
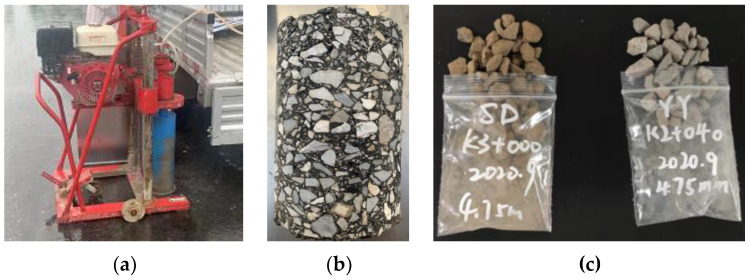
Extraction process of coarse aggregates in the core samples from the asphalt pavement. (**a**) In-situ core-drilling; (**b**) Drilled core sample; (**c**) Coarse aggregates extracted in core sample.

Using a pavement core-drilling machine, as shown in Figure 2, in-situ core-drilling was carried out on the measuring positions where the on-site pavement skid resistance was conducted at different measurement times. The coarse aggregates in core samples were then extracted according to T 0735-2011 in Chinese Specification JTG E20-2011 [33]. The extraction process of coarse aggregates is shown in Figure 2.

Due to the fact that the tires only directly contact with the surface layer of the pavement, the aggregates in other layers are almost unaffected by the wheel wear. Therefore, the exposed coarse aggregates on the surface layer of core samples were taken as test samples that would be subjected to wear caused by vehicle loads, while the coarse aggregates 3~5 cm below the surface layer were collected as the control groups, that is, the aggregates were collected in the original state without being subjected to the vehicle load wear after the pavement was completed and opened to traffic. Coarse aggregates in the control group were obtained from core samples drilled in November 2019.

#### 2.3.3. Measurement of Coarse Aggregate Angularity

(a) Digital image acquisition

**Figure 3:**
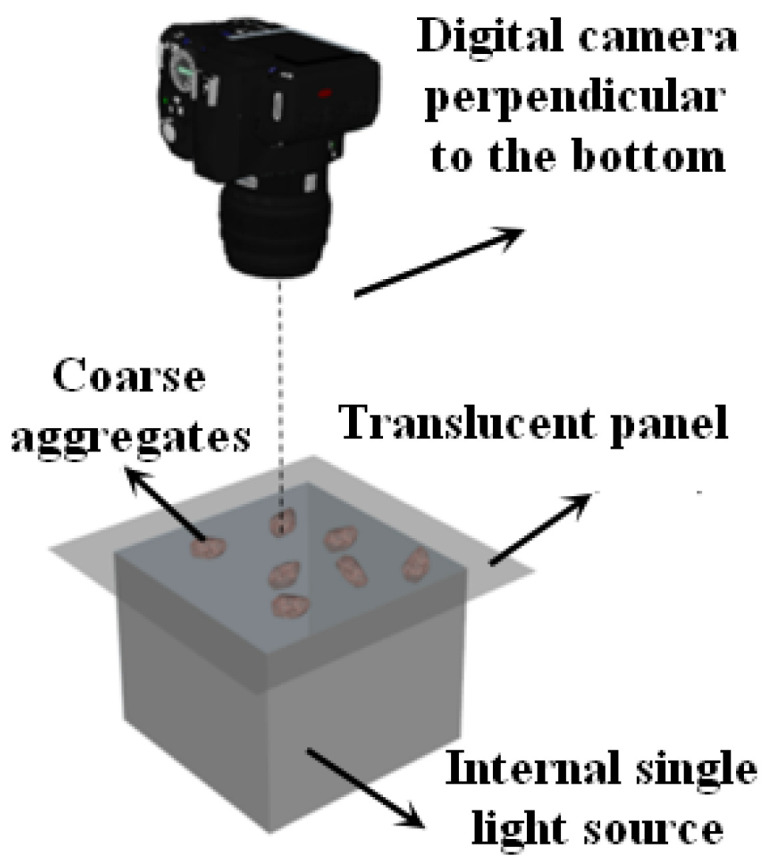
“Backlighting photography” image acquisition system.

The images of coarse aggregates were acquired using a digital camera with 24 million pixels and a self-made “backlighting photography” system. The purpose of adopting the “backlighting photography” is to eliminate the influence of external light sources and obtain high-contrast coarse aggregate images through an internal single light source, so that the profile of coarse aggregates can be accurately identified. The “backlighting photography” image acquisition system is shown in Figure 3.

(b) Digital image processing

**Figure 4:**
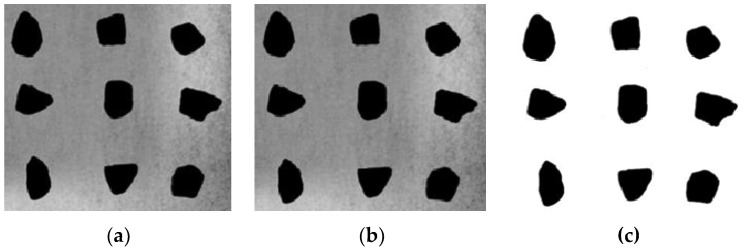
Image processing steps. (**a**) Gray processing; (**b**) Denoising; (**c**) Binarization.

Image-Pro Plus 6.0 (IPP 6.0) software was utilized to process the collected coarse aggregate images. The processing flow was divided into image gray processing, image denoising and image binarization (as shown in Figure 4). Besides, the area, perimeter, major axis length and minor axis length of each aggregate were obtained through the statistical system of IPP. The image processing process is shown in Figure 4.

(c) Angularity evaluation indicators

Relevant research has revealed that convexity and axiality can directly characterize the angularity of aggregates, while roundness can indirectly express the angularity of aggregates by characterizing the degree to which the profile curve of the aggregate surface is close to a circle [34]. Therefore, roundness, convexity and axiality were selected as angularity evaluation indicators based on the calculated parameters using IPP 6.0 software [35]. They were calculated according to Equations (1)–(3), respectively.

(1) $$R=\frac{4\pi A}{{P_{A}}^{2}}$$

where *R* denotes roundness; *A* denotes the area of aggregate images; *P_A_* is the perimeter of aggregate images and *π* denotes the Pi.

(2) $$P=\frac{P_{convex}}{P_{ellipse}}$$

where *P* denotes convexity; *P_convex_* denotes the perimeter of the protrusion and *P_ellipse_* denotes the perimeter of the equivalent ellipse.

(3) $$AS=\frac{L_{maj}}{L_{min}}$$

where *AS* denotes the axiality; *L_maj_* denotes the length of the major axis of the equivalent ellipse and *L_min_* denotes the length of the minor axis of the equivalent ellipse.

#### 2.3.4. Measurement of Coarse Aggregate Micro-Texture

(a) Measurement device

**Figure 5:**
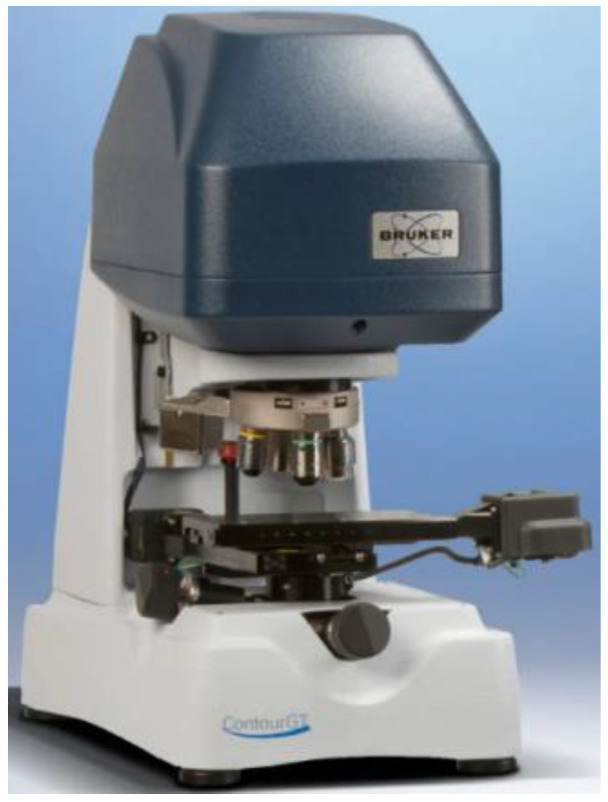
Three-dimensional topography profiler.

The Contour GT-X white light interferometer (as shown in Figure 5) was employed to measure the three-dimensional morphology of coarse aggregates. The interferometer can measure the optical path difference according to the interference principle, based on which relevant physical parameters can be obtained. It is characterized by the fast acquisition of information and high accuracy in morphological information measurement.

(b) Micro-texture measurement method

**Figure 6:**
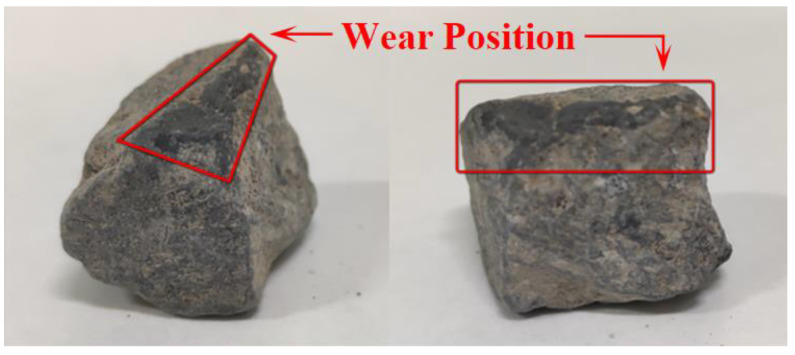
Schematic diagram of coarse aggregate wear position.

Figure 6 is a schematic diagram of the coarse aggregate wear position. The position subjected to wear caused by wheel loads is different due to the differences in the position and shape of aggregates in asphalt mixtures. By observing the surface texture of coarse aggregates after burning and washing, it was found that the worn surface of coarse aggregates subjected to wheel loads for a long time was smooth and dull, which could be clearly distinguished by the naked eye. Therefore, such kind of positions were selected as measurement surfaces, and combined with the measurement requirements of the three-dimensional topography profiler, coarse aggregates with a large wear area and flat wear position were selected from different sizes of aggregates obtained from drilled samples of each test road to measure the aggregate micro-texture.

(c) Micro-texture evaluation indicators

**Figure 7:**
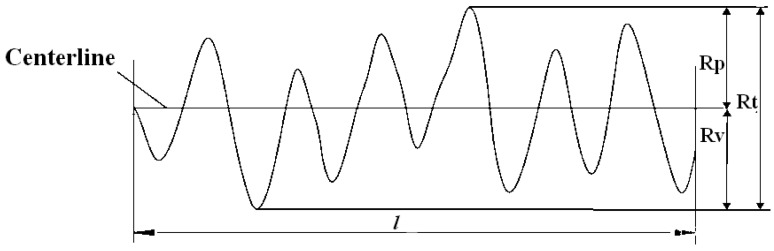
Schematic diagram of the calculation principle of *R_p_*, *R_v_* and *R_t_*.

Research has shown that the maximum profile peak height (*R_p_*), the maximum profile valley depth (*R_v_*) and the total height of the profile (*R_t_*) reflect the height and depth of the profile, while the arithmetic mean deviation (*R_a_*) and the root mean square deviation of the profile (*R_q_*) reflect the dispersion degree of variations in the profile. These five evaluation indicators can effectively characterize the micro-texture of coarse aggregate surfaces [36,37,38,39]. Hence, the five indicators, including the two indicators calculated by Equations (4) and (5) and the three indicators shown in Figure 7, were chosen as the micro-texture evaluation indicators of coarse aggregates. In Figure 7, *R_p_* is the maximum profile peak height within a sampling length; *R_v_* is the maximum profile valley depth within a sampling length and *R_t_* is the total height of the profile, namely the sum of *R_p_* and *R_v_* within an evaluation length.

(4) $$R_{a}=\frac{1}{l}\int_{0}^{1}\left|Z(x)\right|dx$$

where *R_a_* is the arithmetic mean deviation; *l* is the sampling length and Z(x) is the corresponding ordinate value at the *x* coordinate.

(5) $$R_{q}=\sqrt{\frac{1}{l}\int_{0}^{1}Z^{2}(x)dx}$$

where *R_q_* is the root mean square deviation of the profile.

### 2.4. Grey Correlation Entropy Analysis Method

The grey correlation entropy analysis is an improved algorithm based on the grey correlation system theory, which can quantitatively describe and compare the impact degree and contribution of various factors in the system through the correlation degree. In this research, the grey correlation entropy analysis method was employed to analyze the impact of various morphological characteristics of coarse aggregates on the anti-skidding performance of the pavement. The specific calculation steps are as follows [40]:

- Determination of the reference sequence and comparison sequence

The reference sequence *X*_0_ is a data sequence that can reflect the characteristics of the target system, while the comparison sequence *X_i_* is a data sequence composed of factors that affect the system’s behavior. In this research, the skid resistance was regarded as a reference sequence and the results of various indicators of morphological characteristics of coarse aggregates were considered as comparison sequence.

- Dimensionless processing of raw data

To improve the accuracy of the analysis and simplify the calculations, the averaging method was employed to achieve dimensionless transformation of various data sequences.

- Calculation of grey correlation coefficient

The calculation of grey correlation coefficient between each reference sequence and comparison sequence were calculated based on their absolute value, the maximum and minimum value among the absolute values of all the reference sequence and comparison sequence.

- Calculation of distribution density of grey entropy correlation coefficient

The distribution density of the grey entropy correlation coefficient was the ratio of a grey correlation coefficient to the sum of the grey correlation coefficient in the same sequence.

- Calculation of grey entropy correlation degree

The grey entropy correlation degree was calculated based on the distribution density of grey entropy correlation coefficient calculated above.

## 3. Results and Discussion

### 3.1. Attenuation Law of Pavement Skid Resistance

**Figure 8:**
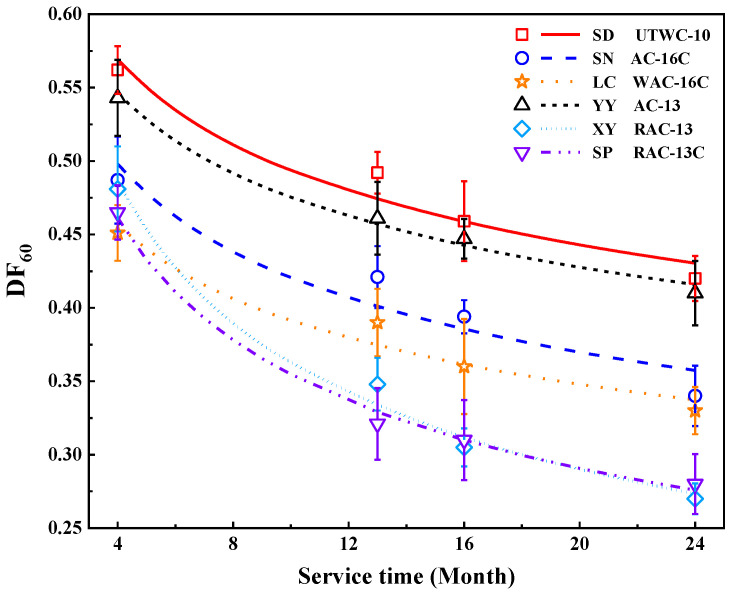
The changes of DF_60_ with service time.

Figure 8 displays the changes of DF_60_ of six roads with the service time. For each measurement position, at least six repeated tests were conducted. In order to analyze the impact of different types of asphalt mixtures on pavement skid resistance, the six roads were divided into UTWC, AC-16 and AC-13 pavement according to their mixture types.

As can be seen from Figure 8, compared with the initial pavement skid resistance, the decline rate of skid resistance of each test road after two years of service varied between 24% and 43%. This is due to differences in pavement materials, mixture design, construction techniques and traffic volume of each test road.

**Figure 9:**
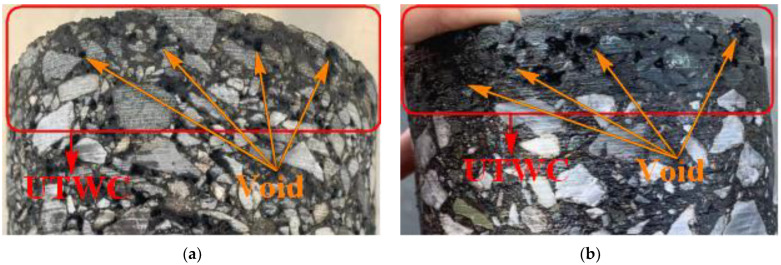
Core samples of ShiDan Road at different service period. (**a**) November 2019; (**b**) September 2021.

Figure 8 also shows that the pavement skid resistance of SD Road maintained a high level throughout the whole service time. This is mainly because the coarse aggregates used in SD Road are basalt, which is different from that (limestone) used in other test roads. Compared with limestone, basalt has higher roughness and wear resistance [10,23], enabling SD Road to still exhibit excellent pavement skid resistance after two years of service, even under the condition of withstanding several times the traffic volume of other test roads. Meanwhile, due to the fact that the initial skid resistance of the newly constructed asphalt pavement after operation is mainly affected by the asphalt film on the pavement surface, the modified asphalt used in SD Road has higher adhesion than the matrix asphalt used in other test roads, thus enhancing the anti-erosion and anti-stripping performance of the asphalt mixture, which has an important impact on slowing down the initial attenuation of pavement skid resistance. In addition, as shown in Figure 9, a high porosity was found in the surface layer of SD Road after two years of service, signifying a good macro-texture depth of SD Road surface [41]. This is because discontinuous gradation is adopted in UTWC pavement, in which coarse aggregates with a particle size above 4.75 mm account for 70%, and its coarse gradation effectively enhances the biting force and internal friction between aggregate particles [8], which contributes to the highest level of pavement skid resistance of SD Road after two years of service to some extent.

As for the attenuation rate of pavement skid resistance of AC-16 pavement, the attenuation rate of pavement skid resistance of SN Road was 21.5% higher than that of LC Road in the whole test period. This is mainly attributed to the fact that LC Road is a secondary highway connecting nearby towns and villages, and its annual average daily traffic is only 5.8% of that of SN Road. In this case, the LC Road suffers less wear from vehicle loads and still has a rich surface texture.

In terms of AC-13 pavement, YY Road exhibited excellent pavement skid resistance, as shown in Figure 8. By the fourth on-site skid resistance test, the attenuation rates of pavement skid resistance of XY Road and SP Side Road were 1.51 and 1.79 times higher than that of YY Road, respectively. This is mainly because RAC-13 is used as the top layer of asphalt pavement on XY Road and SP Side Road. When the asphalt film on the pavement gradually falls off, the aggregates are exposed and directly contact the vehicle, and sufficient friction between tires and pavements cannot be offered by the recycled aggregates with poor angularity and micro-texture, which leads to the continuous and rapid attenuation of pavement skid resistance. Moreover, it was found through investigation that the distance between the construction site of YY Road and the mixing plant is short, with a linear distance of only 2 km, which greatly reduces the temperature loss of hot-mixed asphalt mixture during transportation, thus effectively avoiding the problems of paving and compaction quality caused by construction at low temperature [42] and ensuring the superior and stable texture depth and surface texture of YY Road. This is also the key factor to keep the skid resistance of YY Road at a high level within two years of service.

### 3.2. Variation in Angularity of Coarse Aggregates

**Figure 10:**
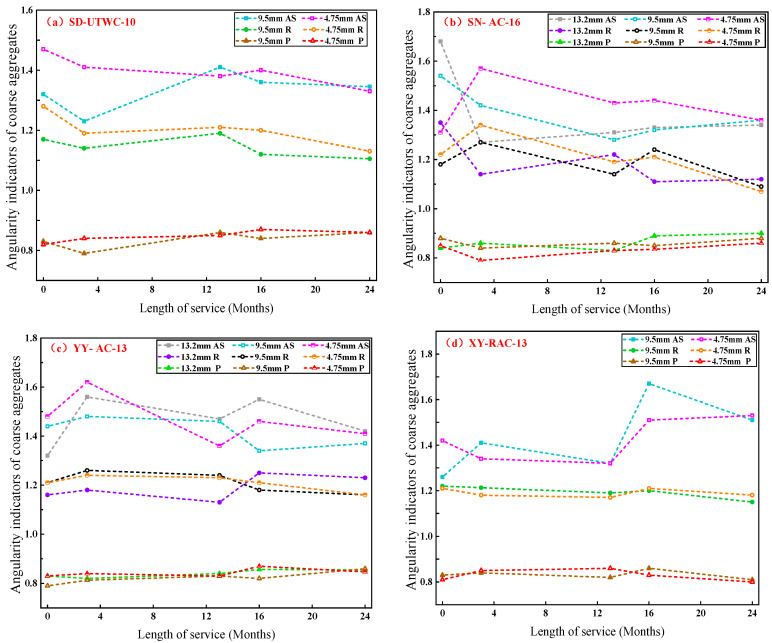
The changes of angularity of coarse aggregates with service time for different roads.

In this study, angularity tests were carried out on coarse aggregates with particle sizes of 4.75~9.5 mm, 9.5~13.2 mm and 13.2~16 mm. It failed to extract coarse aggregates with required particle sizes from some test roads due to the difference in gradation composition of asphalt mixtures, so only coarse aggregates from SD Road, SN Road, YY Road and XY Road were subjected to angularity tests, with the calculation results of the angularity evaluation indicators of the four test roads shown in Figure 10. In Figure 10, similar abbreviations are named in the same way. For example, “13.2 mm *AS*” represents the axiality of coarse aggregates with a particle size of 13.2~16 mm; “9.5 mm *AS*” stands for the axiality of coarse aggregates with a particle size of 9.5~13.2 mm and “4.75 mm *AS*” represents the axiality of coarse aggregates with a particle size of 4.75~9.5 mm.

As shown in Figure 10, among angularity indicators of coarse aggregates, the roundness (*R*) declined overall with the increase in service time. It suggests that the two-dimensional profile of coarse aggregates tends to be round after wearing, with gradually decreased angularity, which results in the decrease in micro-cutting between tires and pavement and the decline of pavement skid resistance.

Figure 10 also shows that there were no obvious change law in convexity (*P*) and axiality (*AS*). It is attributed to the fact that the worn surface of aggregates exposed on the pavement is relatively small during the actual service process. The real wear position of coarse aggregates may not be collected by the “ backlighting photography” system due to the effect of the placement position of coarse aggregates. As a result, the convexity and axiality represented the macro-morphology of coarse aggregates in essence. Therefore, the convexity and axiality of coarse aggregates should not be used separately as evaluation indicators characterizing the attenuation law of coarse aggregate angularity.

### 3.3. Variation in Micro-Texture of Coarse Aggregates

**Figure 11:**
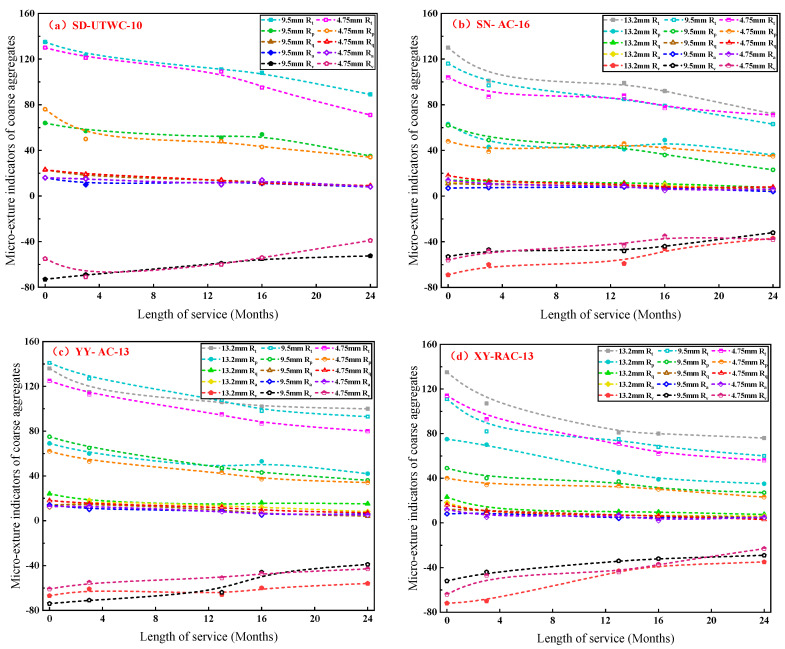
The changes of micro-texture of coarse aggregates with service time for different roads.

Micro-texture measurement was performed on coarse aggregates with particle sizes of 4.75~9.5 mm, 9.5~13.2 mm and 13.2~16 mm for each test road. The micro-texture of coarse aggregates obtained from four roads, SD, SN, YY and XY were measured. Figure 11 shows the calculation results of micro-texture evaluation indicators of the four test roads. In Figure 11, similar abbreviations are named in the same way. For example, “13.2 mm *R_t_*” represents the total height of the profile of coarse aggregates with a particle size of 13.2~16 mm; “9.5 mm *R_t_*” represents the total height of the profile of coarse aggregates with a particle size of 9.5–13.2 mm; and “4.75 mm *R_t_*” represents the total height of the profile of coarse aggregates with a particle size of 4.75~9.5 mm.

As can be seen from Figure 11, during the whole service period, all micro-texture indicators showed a trend of rapid change in the early stage and gradual change in the later stage, which was specifically reflected as the decrease in the maximum profile peak height (*R_p_*) and the total height of the profile (*R_t_*) and the increase in the maximum profile valley depth (*R_v_*). This indicates that with the increase in service time, the worn surface of coarse aggregates tends to be smooth since the asperities on the worn surface of coarse aggregates are gradually worn, and some depressions are filled.

As shown in Figure 11, SD Road with basalt as coarse aggregates still exhibited excellent micro-texture after two years of service, even under the condition of withstanding several times the traffic volume of other test roads. Meanwhile, as shown in Figure 11c,d, compared with YY Road with the same mixture type of AC-13, the attenuation rate of each micro-texture indicator of XY Road was 2~3 times that of YY Road after ten months of service, indicating that when the asphalt film on the pavement falls off, the recycled aggregates with poor angularity and micro-texture are exposed and directly contact with vehicles, resulting in the rapid attenuation of pavement skid resistance. It illustrates that the pavement skid resistance is greatly affected by the type and wear condition of aggregates. By selecting high-quality aggregates with high wear resistance, the initial pavement skid resistance can be improved and its attenuation rate can be reduced.

In addition, the micro-texture indicators of coarse aggregates with different aggregate sizes in the initial state were compared and the results demonstrated that the difference in micro-texture indicators of test coarse aggregates with different particle sizes were all within 10%, as shown in Figure 11b,c, signifying poor correlation between the micro-texture and particle size of coarse aggregates at this stage. However, with the increase in service time, a lower micro-texture attenuation rate was detected in the coarse aggregates with large particle size, indicating that the pavement surface can still maintain excellent skid resistance under long-term wheel loads by increasing the nominal maximum aggregate size or the proportion of coarse aggregates of the mixture.

### 3.4. Correlation between Pavement Skid Resistance and Morphological Characteristics of Coarse Aggregates

The variation tendency of angularity and micro-texture of coarse aggregates is related to the attenuation law of pavement skid resistance. In order to identify the morphological indicators of coarse aggregates which can more accurately characterize pavement skid resistance, the grey correlation entropy analysis method was adopted to compare the influence of each morphological indicator of coarse aggregates on pavement skid resistance. The calculation results of grey correlation entropy between the morphological indicators of coarse aggregates and pavement skid resistance DF_60_ are shown in Table 3.

Table 3 shows that the morphological indicator *R_v_* had the greatest effect on the pavement skid resistance DF_60_, followed by *R_t_*, *R*, *R_q_*, *R_a_*, *R_p_*, *AS* and *P* in turn. The correlation degree between the five micro-texture indicators (including *R_v_*, *R_t_*, *R_q_*, *R_a_* and *R_p_*) and the pavement skid resistance was greater than 0.7, indicating that the micro-texture of coarse aggregates is a crucial factor affecting pavement skid resistance. Among them, the variation range of coarse aggregates micro-texture has the most significant effect on pavement skid resistance, followed by the dispersion degree of variations in the micro-texture. However, among the evaluation indicators of coarse aggregate angularity, only roundness had a correlation over 0.7 with pavement skid resistance, while other angularity evaluation indicators displayed poor correlations with pavement skid resistance.

After asphalt film on the pavement surface disappears due to wear, the vehicle can only contact the worn surface of aggregates on the pavement surface, and pavement skid resistance is ensured by the friction between the tire and pavement provided by aggregate angularity and the micro-texture on the worn surface, as well as rolling friction provided by the pavement macro-texture. With the increasing number of wheel loads, the asperities on the worn surface of coarse aggregates on the pavement surface were gradually worn away, whereas some depressions were filled, manifested as the reduced total height of the profile (*R_t_*) and maximum profile peak height (*R_p_*) and increased maximum profile valley depth (*R_v_*), which represented the variation range of the micro-texture. The variation was directly related to the friction between the tire and pavement, leading to the decline in pavement skid resistance. In addition, the root mean square deviation of the profile (*R_q_*) and the arithmetic mean deviation (*R_a_*) representing the dispersion degree of micro-texture variations were also strongly related to pavement skid resistance, implying that there were some similarities in the wear characteristics of micro-texture of coarse aggregates on the pavement surface. Therefore, compared with the angularity of coarse aggregates, the micro-texture has a greater impact on the skid resistance of actual asphalt pavement. Using aggregates with high roughness can effectively improve pavement skid resistance. However, it is undeniable that this will also increase the rolling resistance of the pavement surface, which will lead to an increase in fuel consumption [43]. Therefore, in the pavement design stage, the morphological characteristics of aggregates should be considered to ensure driving safety while balancing environmental protection and economy.

In terms of the evaluation indicators of coarse aggregate angularity, only the roundness rose under vehicle loads compared with that of aggregates in the initial state, i.e., the exposed aggregates on the pavement surface became more rounded and smooth, whereas the macro-morphological characteristics, namely convexity and axiality, displayed no significant variations. Therefore, the indicators of coarse aggregate angularity are generally not suitable for characterizing the skid resistance of actual asphalt pavement.

## 4. Conclusions

In this research, the skid resistance of six actual roads was observed during the two years to analyze the skid resistance deterioration characteristics. In the meantime, core samples were drilled onsite to investigate the variation of aggregate morphological characteristics of coarse aggregates, including the angularity and micro-texture. Finally, the correlation between the morphological characteristics of coarse aggregates and the pavement skid resistance was established. Based on the test results and analysis, the following conclusions could be drawn.

With the increase in service time, the attenuation of pavement skid resistance gradually slows down, and finally is maintained near a certain value for a long time. Compared with the initial value of pavement skid resistance, the attenuation rate of pavement skid resistance of each test road after two years of service varies between 24% and 43%. The skid resistance of UTWC pavement is better than that of AC pavement, and the skid resistance of new pavement is better than that of recycled pavement. The attenuation rate of pavement skid resistance can be slowed down by increasing the proportion of coarse aggregates, using coarse aggregates with a large particle size, or using basalt as coarse aggregates.

With the increase in service time, the coarse aggregate angularity indicator roundness (R) decreases overall, with a decline of 15 percent, while convexity (*P*) and axiality (*AS*) have no obvious variation law. In the actual service process of pavement, the two-dimensional profile of coarse aggregates tends to be round, with angularity gradually decreasing. Therefore, the convexity and axiality of coarse aggregates should not be used as evaluation indicators characterizing the attenuation law of aggregate angularity alone.

With the increasing service time, the asperities on the surface of coarse aggregates gradually wear away, and the surface of coarse aggregates tends to be smooth, which is manifested by the decrease in the maximum profile peak height (*R_p_*) and the total height of the profile (*R_t_*) and the increase in the maximum profile valley depth (*R_v_*). The wear rate of micro-texture can be effectively slowed down by selecting coarse aggregates with high roughness and high wear resistance so that pavement skid resistance can keep a high level for a long time.

The grey correlation entropy between all the micro-texture indicators and angularity indicator roundness of coarse aggregates and skid resistance is more than 0.7, while the other angularity indicators have poor correlations with pavement skid resistance. It signifies that among the evaluation indicators of morphological characteristics of coarse aggregates, the micro-texture affects pavement skid resistance more greatly, while angularity indicators of coarse aggregates are generally not suitable for characterizing the skid resistance of actual asphalt pavement.

## 5. Future Research

Skid resistance attenuation of different types of asphalt pavements through long-term tracking on actual roads was explored and its correlation with morphological characteristics of coarse aggregates in the surface layer was analyzed. In future research, more roads with different materials compositions will be included in the investigation. We will explore the similarities and differences between the attenuation characteristics of skid resistance and the surface texture of asphalt pavement by combining the on-site investigation and laboratory simulation, which contribute to the improvement of skid resistance and determination of pavement maintenance time.

## Figures and Tables

**Table 1:** Basic information of test roads.

Road Name	SD	SN	LC	YY	XY	SP
Road type	New road	Major repair	Major repair	Upgrading	Major repair	Upgrading
Road class	Trunk	Secondary	Secondary	Trunk	Secondary	Trunk
Sample length/km	13.00	2.00	9.70	1.65	5.00	6.32
Number of lanes	6	4	2	4	2	8
Opening time	August 2019	Octobr 2019	August 2019	August 2019	Octobr 2019	August 2019
Annual average daily traffic volume/vehicle	27,389	8500	495	6350	5630	11,663

**Table 2:** Type and design information of asphalt mixture of test road sections.

Road Name	SD	SN	LC	YY	XY	SP
Mixture type in the surface layer	UTWC-10	AC-16C	WAC-16C	AC-13	RAC-13	RAC-13C
Asphalt type	Modified	70#	90#	70#	70#	70#
Coarse aggregate type	Basalt	Limestone	Limestone	Limestone	Limestone	Limestone
Nominal maximum aggregate size/mm	9.5	16	16	13.2	13.2	13.2
Coarse aggregate ratio/%	70	54.5	55.2	55.8	49.7	47.3
Coarse aggregate crushing value/%	13.2	15.1	13.3	16.3	18.6	17.5
Coarse aggregate Los Angeles wear value/%	14.3	18.5	14.1	14.1	18.2	16.4
Asphalt-aggregate ratio/%	4.8	4.6	4.5	4.8	4.8	4.8
Voids/%	13.8	4.8	5.4	4.5	4.6	4.3

Notes: Similar abbreviations use the same naming method. RAC-13 denotes Recycled Asphalt Concrete with a nominal maximum aggregate size of 13.2 mm; AC denotes Asphalt Concrete; WAC denotes Warm Asphalt Concrete; UTWC denotes Ultra-Thin Wearing Course.

**Table 3:** Grey correlation entropy between morphological characteristics of coarse aggregates and skid resistance.

Morphological Indicators of Coarse Aggregates	*R_a_*	*R_p_*	*R_q_*	*R_t_*	*R_v_*	*P*	*AS*	*R*
Correlation degree	0.715	0.705	0.728	0.747	0.752	0.668	0.671	0.731

## Data Availability

All data, models, and codes generated or used in this study are included in the submitted manuscript.
